# Classification of Histologically Proven Inflammation in Clinically Inactive Corneal Scars: Implications for Graft Outcomes After Penetrating Keratoplasty

**DOI:** 10.3390/jcm14020461

**Published:** 2025-01-13

**Authors:** Max Schliffka, Helena Siegel, Claudia Auw-Haedrich, Christian van Oterendorp, Daniel Boehringer, Thomas Reinhard

**Affiliations:** 1 Eye Center, Faculty of Medicine, University of Freiburg, 79106 Freiburg im Breisgau, Germany; max@schliffka.de (M.S.); helena.siegel@uniklinik-freiburg.de (H.S.); daniel.boehringer@uniklinik-freiburg.de (D.B.); thomas.reinhard@uniklinik-freiburg.de (T.R.); 2 Faculty of Medicine, University of Freiburg, 79110 Freiburg im Breisgau, Germany; 3 Eye Clinic, University Hospital Basel, 4031 Basel, Switzerland; 4 Department of Ophthalmology, University Medical Center, 37085 Göttingen, Germany; christian.oterendorp@med.uni-goettingen.de

**Keywords:** corneal scar, corneal inflammation, herpetic keratitis, inflammation score, corneal graft rejection, corneal graft failure

## Abstract

**Background/Objectives**: Clinically inactive corneal scars have repeatedly been shown to exhibit histological inflammation. This study aimed to evaluate the degree of histological inflammation in clinically inactive corneal scars of different origins and its correlation with graft rejection and failure following penetrating keratoplasty. **Methods**: The study included 205 primary corneal explants with clinically inactive central scars resulting from herpes simplex virus keratitis (HSV, *n* = 55), keratoconus (*n* = 39), mechanical trauma (*n* = 27), scrophulosa (*n* = 22) or other/unknown causes (*n* = 62). Central histological sections were categorized by the degree of inflammation, and an overall inflammation score (IS) was calculated. **Results**: HSV-associated scars exhibited a trend towards more graft rejection with higher IS (*p* = 0.074). Keratoconus-associated scars showed no IS-dependent differences in graft rejection or failure. The rejection rate in this group was 13/39. Scars resulting from mechanical trauma, such as perforating injuries, demonstrated a trend towards higher graft rejection (*p* = 0.15) and failure rates (*p* = 0.089) with increasing IS. The rejection rate in this group was 11/27. Scrophulosa-associated scars had significantly higher graft rejection rates (*p* = 0.041) at a lower cut-off of 0.06 compared to the cut-off of 0.36 for the other groups. Scars of other or unknown causes showed no IS-dependent differences in graft rejection or failure. **Conclusions**: Histological inflammation in HSV scars and scars resulting from mechanical trauma appeared to contribute to graft rejection. Despite low IS, the rejection rate in keratoconus scars and scars following mechanical trauma was unexpectedly high, indicating the presence of other influencing factors. While some correlations did not reach statistical significance due to small sample sizes in the subgroups, the observed trends should be considered clinically relevant. The study may have been “underpowered”, as histopathologically inflamed specimens with clinically inactive corneal scars are relatively rare.

## 1. Introduction

A number of studies [1,2,3] along with our own experience at the Ophthalmopathology Laboratory of the Freiburg Eye Center indicate that clinically inactive corneal scars often exhibit histological inflammation. This study aimed to quantify these subclinical inflammations and analyze their potential impact on complications following penetrating keratoplasty (PK). We focused on scars resulting from herpes simplex keratitis, but also included scars associated with keratoconus, mechanical trauma, scrophulosa and scars of other/unknown origin for comparison. Scrophulosa corneal scars present as pale gray, large-area corneal opacities, often caused by keratoconjunctivitis scrophulosa, a hyperergic fulminant inflammatory reaction to microbial antigens. Histologically, these scars exhibit increased stromal accumulation of AMP-positive material, likely glycosaminoglycans.

Notably, trachoma, the most common reason for corneal scars, was not represented in our cases. Worldwide, approximately 10–12 million people suffer from corneal scars, and around 250,000 are blind as a result [4].

We were particularly interested in the potential influence of histologically detectable inflammation on the major complications of penetrating keratoplasty, including graft rejection, and herpes simplex virus (HSV) keratitis recurrence in the case of underlying herpetic disease and graft failure.

A study by Shtein et al. (2008) [2], which investigated the impact of histologically detectable inflammation in active and inactive herpetic corneal scars on the complications of graft rejection, HSV recurrence and graft failure, found a correlation with graft rejection, thus providing initial indications.

A possible association between histological inflammation and the postoperative clinical course could complement established risk factors such as the underlying disease [5], preoperative corneal vascularization [6,7,8,9,10], or lack of HLA (human leucocyte antigen) compatibility [5,11,12,13,14]. This could contribute to the selection and dosage of prophylactic drug therapies, particularly virostatic and immunosuppressive treatments [15,16,17,18,19,20,21].

The aim of this study was to develop and apply a quantitative inflammation score to clinically inactive corneal scars of various origins, with a particular focus on HSV keratitis. We evaluated the relationship between histological inflammation and post-PK complications such as graft rejection, graft failure and HSV recurrence, aiming to improve postoperative care strategies and optimize graft survival through more individualized management.

## 2. Materials and Methods

### 2.1. Patients and Clinical History

Our study was conducted retrospectively. We included patients who underwent penetrating keratoplasty due to a clinically inactive corneal scar between 1 January 1998 and 31 December 2020 at the Freiburg Eye Center or between 1 January 2017 and 31 December 2020 at the Department of Ophthalmology of the University Medical Center Göttingen. All patients were at least 18 years old at the time of keratoplasty and all corneal explants were examined at the Ophthalmopathology Laboratory at the Freiburg Eye Center.

A total of 234 cases were initially eligible, with 29 excluded—18 due to primary repeat keratoplasty, 7 due to inadequate or missing patient records, 3 due to missing histological slides and 1 case due to self-inflicted corneal trauma.

The final cohort consisted of 205 patients, 188 from Freiburg and 17 from Göttingen. A total of 55 corneal scars were of herpetic origin, 39 were due to keratoconus, 27 were associated with mechanical trauma, 22 were associated with scrophulosa and 62 were of other or unknown origin. The latter scars were, for example, of infectious or dystrophic origin or caused by chemical or thermal influences.

Of the 55 patients included, 21 were female and 34 were male. The average patient age at the time of penetrating keratoplasty was 60 ± 15 years (range: 21–87 years); the average follow-up time was 3.8 ± 3.6 years (range: 0.8–15.4 years).

Patient data were retrospectively extracted from medical records, including the pre- and postoperative medication as well as events of transplant rejection, HSV recurrence and graft failure.

Immediately postoperatively, 47 patients were treated with oral acyclovir (5 × 200 mg/d–5 × 400 mg/d) or valaciclovir (3 × 500 mg/d), 12 with oral mycophenolate mofetil (2 × 1 g/d), while 35 patients were treated with oral steroids (methylprednisolone 16–60 mg/d). All patients received steroid eye drops.

For graft rejection, standard clinical signs were considered such as epithelial rejection lines, subepithelial infiltrates and endothelial cell precipitates indicative of an endothelial rejection line as well as associated graft edema and anterior uveitis [22] (pp. 647–648). Ambiguous statements such as “suspected creeping endothelial rejection” were evaluated as graft rejection in case of doubt.

HSV recurrence was classified when suspicion was documented in the records. If graft rejection or HSV recurrence were mentioned as differential diagnoses, the presence of both was assumed.

Graft failure was defined as irreversible graft opacification. In cases of ambiguity regarding visual acuity reduction, a decrease of 0.2 logarithmic units was used as the threshold to classify graft failure [23].

### 2.2. Histology

All corneal explants were processed and stained using hematoxylin and eosin (HE) and periodic acid–Schiff (PAS). The histological evaluation was performed according to standardized protocols [24], and the corneal sections were digitized for evaluation und further scoring using a slide scanner.

The histological assessment in our study was carried out in two independent steps by a certified ophthalmopathologist (CAH, head of the Ophthalmopathology Laboratory) and a senior ophthalmologist with five years of experience in ophthalmic pathology (HS), along with a medical doctoral candidate (MS).

### 2.3. Inflammation Scoring

**Figure 1:**
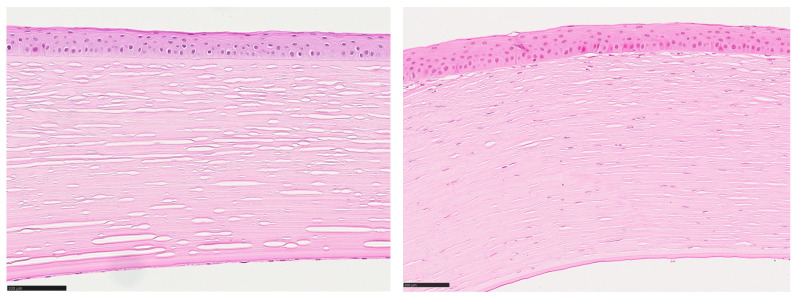
**Left**: HE-stained reference section for a corneal section without inflammation. No inflammatory cells are visible. **Right**: HE-stained reference section for a corneal section with “mild inflammation”. Some inflammatory cells are visible. Magnification bar = 100 µm.

**Figure 2:**
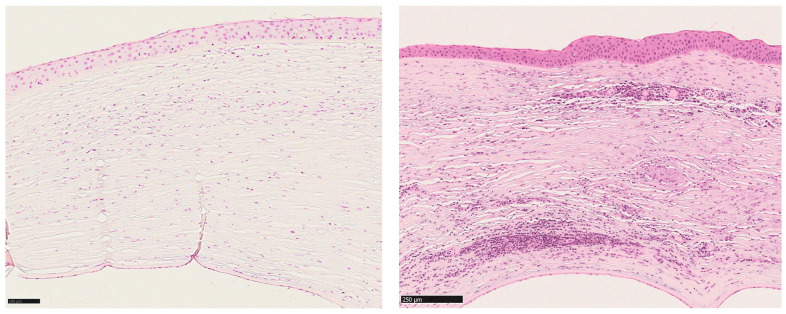
**Left**: HE-stained reference section for a corneal section with “moderate inflammation. Numerous inflammatory cells are visible. **Right**: HE-stained reference section for a corneal section with “severe inflammation”. The stroma is heavily infiltrated by inflammatory cells. Magnification bar = 100 µm on the left and 250 µm on the right.

To quantify the degree of inflammation, we developed an inflammation score by defining 4 reference sections of corneal specimens, each representing one of 4 degrees of severity of inflammation (Figure 1 and Figure 2). The definition of these 4 reference sections was based on the work of Shtein et al. (2008) [2], who assigned one of 4 degrees of severity (“none visible”, “mild”, “moderate” and “severe”) to the entire cornea.

**Figure 3:**
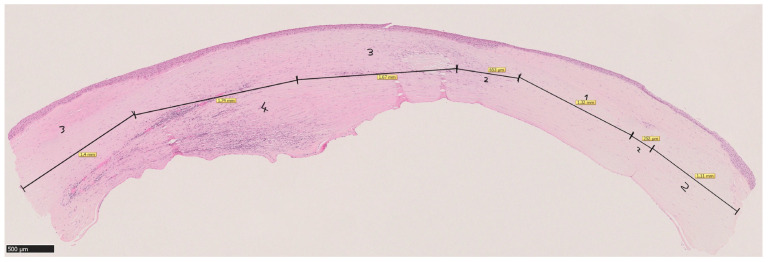
An example of an HE-stained corneal section divided into sectors of equal inflammation severity. 1 = “no inflammation”, 2 = “mild inflammation”, 3 = “moderate inflammation”, 4 = “severe inflammation”. Magnification bar = 500 µm.

Unlike Shtein et al., who assessed inflammation as a single score for the entire cornea, our approach was novel in that we analyzed individual sectors of each corneal section which contained different inflammation intensities and their respective proportions of the entire length of the corneal section (Figure 3). The hypothesis was that this approach would allow for a more detailed and comprehensive assessment of the spatial distribution of inflammatory activity across the corneal tissue.

Whenever necessary, the four- or six-eyes principle (MS, HS, CAH) was used to categorize ambiguous corneal areas within the histological sections. For each cornea, we determined the percentage of the respective inflammation level in relation to the length of the entire analyzed corneal section. We then calculated an overall score (inflammation score, IS) from the four inflammation percentages available for each cornea. This score roughly corresponds to an integration of the inflammation severity over the length of the cornea. To achieve this, we assigned weighting factors to the four levels of inflammation based on our reference images, resulting in greater weight being given to more severe inflammations in our score. The inflammation score can be expressed with the following formula:

Inflammation score (IS)=A×a+B×b+C×c+D×d

where

A: Weighting factor for “no inflammation” = 0.

B: Weighting factor for “mild inflammation” = 1.

C: Weighting factor for “moderate inflammation” = 3.

D: Weighting factor for “severe inflammation” = 15.

a: Proportion of corneal length with “no inflammation”.

b: Proportion of corneal length with “mild inflammation”.

c: Proportion of corneal length with “moderate inflammation”.

d: Proportion of corneal length with “severe inflammation”.

### 2.4. Rationale for Weighting Factors

We determined the weighting factors based on the inflammation density of our reference sections as follows: A = 0; B = 1; C = 3 and D = 15. These numbers were chosen based on the observation that a corneal section classified as “moderately inflamed” contains approximately three times as many inflammatory cells as a section classified as “mildly inflamed”. Similarly, the density of inflammatory cells in a section classified as “severely inflamed” is about five times higher than in a section classified as “moderately inflamed” (D = C × 5 = 15).

These weighting factors reflect the exponential increase in inflammatory cell density between mild, moderate and severe inflammation, ensuring that even small areas of severe inflammation significantly impact the overall score. According to this system, the inflammation score for a given section can range from 0 to 15, depending on the proportions of each inflammation level observed.

### 2.5. Statistical Methods

Statistical analyses were performed using R Studio software version 4.3.2. Descriptive statistics were calculated, and associations between histological inflammation and clinical outcomes (graft rejection, graft failure and HSV recurrence) were analyzed using the log-rank test, chi-square test, and Kaplan–Meier survival curves. Additionally, histograms were created. A *p*-value of <0.05 was considered statistically significant.

## 3. Results

### 3.1. HSV-Associated Corneal Scars (n = 55)

**Figure 4:**
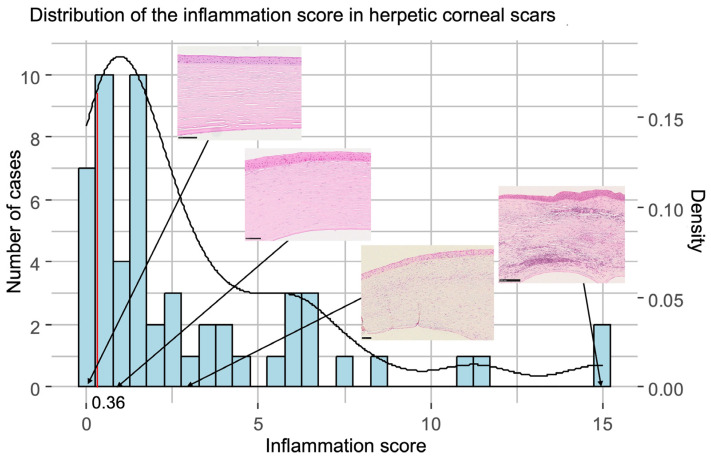
Distribution of the inflammation score in the cases of herpes simplex virus keratitis (HSV)-associated corneal scars. The black curve indicates a continuous estimate of the distribution of our inflammation score in the population (method: kernel density estimation). The arrows from the corneal sections (reference images from subsection “Evaluation of inflammation”) show the extent to which a cornea would have to be inflamed over its entire length in order to obtain the corresponding value of our score. The red line at 0.36 marks the point at which a cornea is considered to be “inflamed” overall.

Scars of herpetic origin were histologically more inflamed than scars of other origins (Figure 4). The inflammation score ranged from 0 (no inflammatory cells) to 15 (intense inflammation) with a median score of 1.60 and a mean of 3.09.

In the cases of herpetic corneal scars, 13 graft rejections, 14 HSV keratitis recurrences and 4 graft failures were noted. The median times of occurrence were 0.8, 2.4 and 2.4 years, respectively (ranges: 0.17–5.90, 0.17–15.26 and 1.31–4.36 years).

### 3.2. Influence of Histologically Proven Inflammation on the Rate of Postoperative Complications

Following the methodological approach of Shtein et al. (2008) [2], we first divided our patients into four groups according to the severity of inflammation histologically detected in their corneal explants (“no inflammation”, “mild inflammation”, “moderate inflammation” and “severe inflammation”). For this classification, we defined a baseline value of our inflammation score (IS) of 0.36, as explained below. Corneal explants with an IS < 0.36 were classified as “non-inflamed” (group 1). For the “inflamed” groups, the cut-offs were calculated using the weighting factors (A = 0, B = 1, C = 3, D = 15):

- IS ≥ 0.36 and <1.08 (= 0.36 × C) was “mild inflammation” (group 2);
- IS ≥ 1.08 and <5.40 (= 0.36 × D) was “moderate inflammation” (group 3);
- IS ≥ 5.40 was “severe inflammation” (group 4).

**Figure 5:**
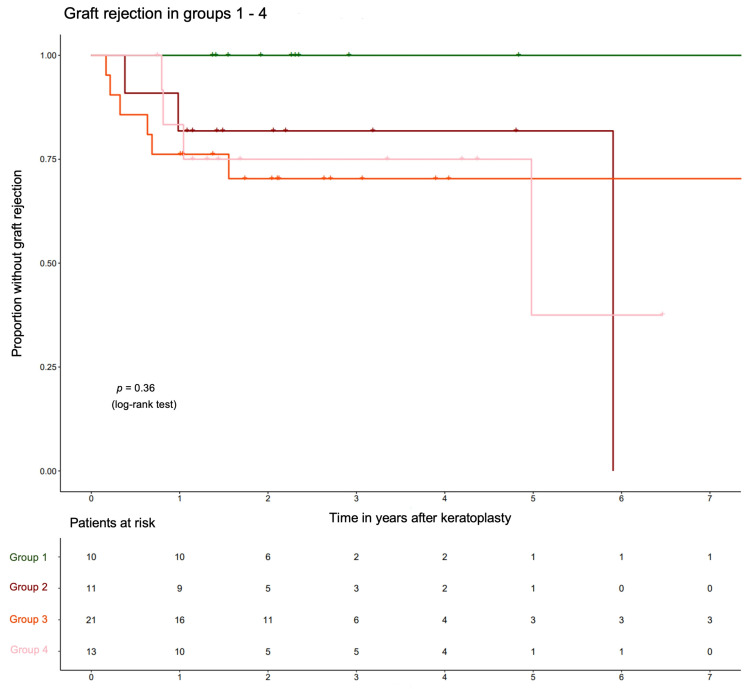
Kaplan–Meier curves for the occurrence of graft rejection in groups 1–4 in the cases of HSV-associated corneal scars. The groups are defined in subsection “Influence of histologically proven inflammation on the rate of postoperative complications” (group 1: “no inflammation”, group 2: “mild inflammation”, group 3: “moderate inflammation”, group 4: “severe inflammation”).

This classification results in the Kaplan–Meier curves shown in Figure 5 for graft rejection as an endpoint.

The Kaplan–Meier curves for graft rejection (Figure 5) showed that all 13 transplant rejections occurred in the inflamed groups, justifying the baseline IS of 0.36. Since the curves for “mild,” “moderate” and “severe” inflammation did not significantly differ (*p* = 0.99, log-rank test), it seemed reasonable to combine these groups.

**Figure 6:**
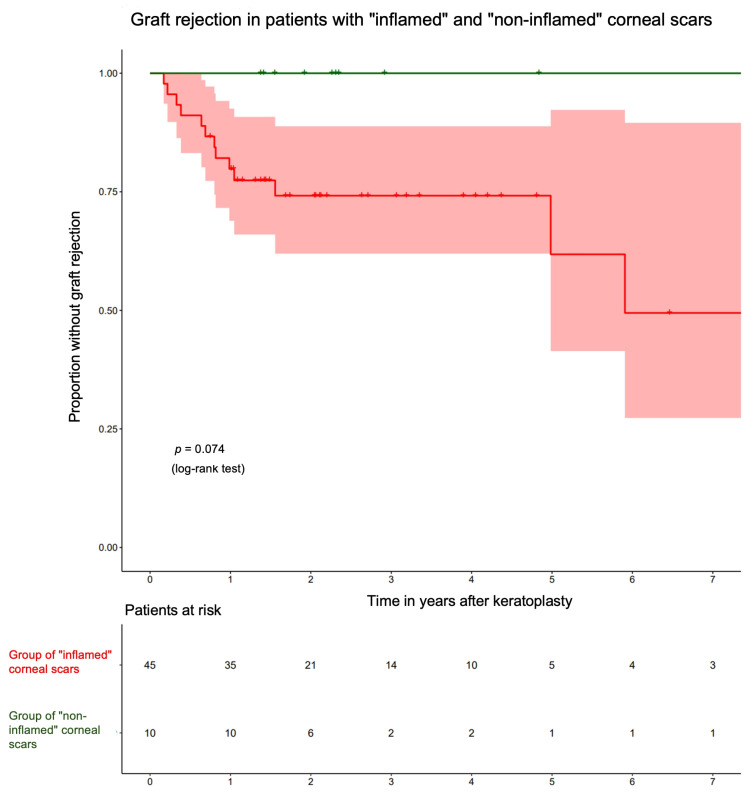
Kaplan–Meier curves for the occurrence of graft rejection in the groups of “inflamed” and “non-inflamed” herpes simplex virus keratitis (HSV)-associated corneal scars. The red-colored area surrounding the Kaplan-Meier curve represents its 95% confidence interval.

While the Kaplan–Meier curves (Figure 6) do not show a statistically significant difference (*p* = 0.074, log-rank test, *n* = 55), there is a noticeable trend indicating that patients with “inflamed” corneal explants are more frequently affected by graft rejection over time, in contrast to those with “non-inflamed” explants. The latter group did not experience any graft rejection in our dataset. The calculated minimum sample size to achieve a 95% confidence level is approximately 65 cases.

Dividing our patients into four groups did not yield meaningful results for any of the other endpoints, so subsequent analyses used only two groups (“inflamed” vs. “non-inflamed”).

While there is a trend toward graft rejection in “inflamed” corneal explants, no such trend is observed for HSV recurrence (*p* = 0.91, log-rank test) or graft failure (*p* = 0.66, log-rank test).

### 3.3. Keratoconus-Associated Scars (n = 39)

Scars of non-herpetic origin were significantly less inflamed overall. For keratoconus scars, inflammation scores ranged between 0.0 and 0.99, with a median value of 0.1 and a mean of 0.2. Using the same cut-off value of 0.36 as for herpetic scars, we found no significant differences between “inflamed” (*n* = 6) and “non-inflamed” (*n* = 33) corneal explants with regard to our endpoints of graft rejection and graft failure (*p* > 0.65 in each case, log-rank test). Surprisingly, we observed a total of 13 graft rejections and only one graft failure.

The majority of graft rejections (11 out of 13) and the single graft failure occurred in the “non-inflamed” corneal explants group.

### 3.4. Scars Associated with Mechanical Trauma (n = 27)

Scars associated with mechanical trauma were moderately inflamed with inflammation scores ranging from 0 to 4.92 (median: 0.26 and mean: 0.47). We observed eleven graft rejections and seven graft failures.

Using our cut-off value of 0.36, both graft rejections and graft failures were more frequent in the “inflamed” corneal scars group (*n* = 9 of 27 cases). However, no statistical significance was achieved (*p* = 0.15 for graft rejection and *p* = 0.089 for graft failure, log-rank test). The graft rejection rate and graft failure rate were 56% (5 cases) and 44% (4 cases), respectively, in the “inflamed” corneal explants group. In comparison, the rates in the “non-inflamed” corneal explants group were 33% (6 cases) and 17% (3 cases).

### 3.5. Scrophulosa-Associated Scars (n = 22)

Scrophulosa-associated scars were very mildly inflamed with inflammation score values between 0 and 0.71 (mean: 0.065, median: 0.0). Due to the mild inflammation, our cut-off value of 0.36 did not allow us to form meaningful groups.

However, lowering the cut-off value to 0.06 seems to allow a clearer distinction between “inflamed” (*n* = 7) and “non-inflamed” (*n* = 15) corneas.

We observed four graft rejections and two graft failures. Three of the four graft rejections occurred in the “inflamed” corneas group, and the Kaplan–Meier curves of the two groups differ significantly from each other (*p* = 0.041, log-rank test). One graft failure occurred in both groups.

### 3.6. Scars of Other/Unknown Origin (n = 62)

The inflammation score of the scars of other or unknown origin ranged between 0 and 4.34 (median: 0.15, mean 0.57).

A total of thirteen transplant rejections and four transplant failures occurred. In the “inflamed” group (*n* = 22, IS ≥ 0.36), graft rejection occurred in 18% (4 cases) with no graft failures. In the “non-inflamed” group (*n* = 40, IS < 0.36), graft rejection occurred in 23% (nine cases) and graft failure in 10% (four cases).

In this very heterogeneous group, we did not find any significant differences with regard to our endpoints between the cases with histologically “inflamed” corneal explants (*n* = 22, with an inflammation cut-off score of 0.36) and those with “non-inflamed” corneal explants (*n* = 40) (*p* > 0.2 each, log-rank test).

## 4. Discussion

### 4.1. Classification of Histologic Inflammation

Our results confirm that clinically inactive corneal scars often exhibit histologically detectable inflammation, particularly in HSV-associated scars, while scars associated with mechanical trauma showed moderate levels, and keratoconus and scrophulosa-associated scars showed low levels of inflammation [1,2,3].

To our knowledge, no prior study has classified subclinical inflammation in these scars as accurately using an inflammation score derived from individual corneal segments. Despite some variability in the results, our findings align with previous reports, such as those by Hogan et al. (1964) [1] and Knöbel et al. (1981) [3]. The distribution of inflammation severity in herpetic scars is comparable to Shtein et al. (2008), even though their study included clinically active cases [2].

### 4.2. Herpes Simplex Virus Keratitis (HSV)-Associated Scars

Shtein et al. (2008) [2] analyzed 62 corneal explants of herpetic origin (60 of them with scars, 12 showing clinical activity) to assess the extent of histological inflammation and its influence on postoperative complications (graft rejection, HSV recurrence and graft failure) after penetrating keratoplasty. For this purpose, they divided the corneal explants into four groups based on the severity of inflammation: “none visible”, “mild”, “moderate” and “severe”. Of the 62 corneal explants, 16 were classified as “none visible” (26%), 12 as “mild” (19%), 15 as “moderate” (24%) and 19 as “severe” (31%).

In our collection of 55 clinically inactive herpetic corneal scars, 10 were classified as non-inflamed (18%), 11 as mildly inflamed (20%), 21 as moderately inflamed (38%) and 13 as severely inflamed (24%). The inclusion of all the corneal sectors of the respective corneal sections into the scoring of each case allowed a more precise classification into the various inflammation categories. Nevertheless, the percentage of each inflammation category in our clinically inactive scars is somehow comparable with that of Shtein et al., although 12 of their herpetic scars were clinically active. Their 62 cases contained more nonvisible (26%) and severe inflammation (31%) and less moderate inflammation (24%), which might reflect the variability of inflammation in clinically inactive herpetic scars.

In herpetic corneal scars, histologically detectable inflammation, but not its severity, was associated with graft rejection, although not statistically significant (*p* = 0.074). All 13 rejections occurred in inflamed corneas.

This trend aligns with Shtein et al. (2008) [2], who reported a significant correlation between histologically inflamed corneal explants and the graft rejection rate (*p* = 0.02, log-rank test) while the severity of inflammation did not seem to play a role. The inclusion of clinically active cases in their study (12 of 62 cases) suggests that the effect of histological inflammation alone may be less pronounced. Nonetheless, our findings indicate that histologically bland herpetic scars could be considered lower risk, possibly requiring less aggressive immunosuppression.

It is possible that our number of cases was not sufficient to demonstrate a significant correlation between histologically detectable inflammation and graft rejection. Due to the rarity of the disease, future studies with a multicenter approach should be conducted to acquire a sufficient number of cases.

Like Shtein et al. (2008), we found no significant association between histologically detectable inflammation and HSV recurrence or graft failure.

HSV keratitis is considered a risk factor for graft rejection and failure following penetrating keratoplasty [5,25,26,27,28]. Surprisingly in our study, HSV-associated scars did not show a significantly higher rate of graft rejection and failure compared to the other groups (scars associated with keratoconus, mechanical trauma or other/unknown reason), despite having a higher inflammation score. Clinically inactive herpetic corneal scars may therefore represent a subgroup of HSV cases requiring transplantation that have a better overall prognosis despite a histologically proven inflammation in comparison to scars of other origin.

### 4.3. Scars in Keratoconus

Although associations with molecular inflammatory mediators have been repeatedly demonstrated in recent years, keratoconus is traditionally considered a non-inflammatory disease [29,30,31,32,33,34,35,36]. Therefore, histologically detectable inflammation is surprising. Using the same cutoff as for the herpes scars of 0.36, we found six inflamed and thirty-three non-inflamed cases with a significantly lower mean inflammatory score of 0.2 in contrast to 3.09 of the herpes-associated scars. Mechanical irritation, such as from contact lenses, might explain the inflammation [37], resulting in a central scar that restricts vision, which was present in all our cases.

In contrast to the herpetic corneal group, no trend in graft rejection differences was observed between inflamed and non-inflamed keratoconus scars (*p* = 0.65, log-rank test).

Surprisingly, we recorded 13 graft rejections (11 of the “non-inflamed” group) among the keratoconus cases, which are classified in the literature as low-risk with regard to keratoplasty [38,39,40,41].

This could be due to the more frequent ophthalmologic follow-ups in keratoconus patients due to their bilaterally progressive disease [29], leading to the earlier detection of mild rejection episodes.

It is also conceivable that the scar subgroup in keratoconus is associated with frequent graft rejection. However, the thirteen graft rejections in our patient population do not affect the overall prognosis with only one graft failure occurring.

### 4.4. Scars in Association with Mechanical Trauma

Scars from mechanical trauma showed a trend towards higher rates of graft rejection (*p* = 0.15) and failure (*p* = 0.089) in inflamed corneas, although the results were not statistically significant. Interestingly, the rate of graft rejections (eleven out of twenty-seven cases) and graft failures (seven out of twenty-seven cases) was higher in this group compared to other diagnostic groups. These observations warrant further investigation, as no comparable studies exist to date.

### 4.5. Scrophulosa-Associated Scars

Scrophulosa scars exhibited significantly lower levels of inflammation. To create two usable comparison groups, we lowered the inflammation score cut-off to 0.06 for scrophulosa-associated scars, revealing a significant difference in graft rejection rates between the “inflamed” and “non-inflamed” groups (*p* = 0.041, log-rank test). However, these findings should be interpreted with caution due to the small sample size (*n* = 22) and the unproven reliability of our inflammation score in the low range (0.0 to 0.1). Further research with larger sample sizes and improved inflammation characterization is needed to elucidate the role of histological inflammation in these cases.

### 4.6. Scars of Other/Unknown Origin

In the heterogeneous diagnostic group of “scars of other/unknown etiology,” no significant differences were found between histologically “inflamed” and “non-inflamed” corneal explants regarding our endpoints. The diversity of underlying pathologies prevents meaningful generalization of the results or direct comparison with HSV keratitis cases.

### 4.7. Other Factors Influencing Rejection

In addition to the risk factors mentioned in the introduction, such as the extent of preoperative clinical corneal vascularization and the HLA compatibility between donor and recipient, the following factors could further increase the risk of graft rejection in addition to inflammation: keratoplasty in the fellow eye, young donor age, high graft endothelial cell density, short duration of corticosteroid application, longer time from keratoplasty to complete suture removal, suture-associated complications [42] and short death-to-surgery time [43] could increase the risk of graft rejection in addition to inflammation. It is inconclusive whether young recipient age and female gender are associated with higher rejection rates following keratoplasty [42].

Regarding the statistical evaluation of this possible correlation in our herpetic scar group, an age- and gender-adjusted analysis using Cox regression was not possible. This is because we did not observe any cases of graft rejection in the group of “non-inflamed” corneal explants of herpetic corneal scars and therefore could not form a hazard ratio from the hazards of the “non-inflamed” and “inflamed” corneal explants groups. The size of the other groups was too small for the Cox regression analysis.

### 4.8. Limitations of the Study

Limitations of the study include the retrospective analysis of clinical data and the small sample size, which, due to the absence of rejection cases in the non-inflamed group of herpetic scars, prevented a Cox regression analysis that could account for other possible risk factors.

## 5. Conclusions

Histological inflammation in HSV- and mechanical trauma-associated scars appears to increase the risk of graft rejection. Despite low inflammation scores, keratoconus-associated scars and scars after mechanical trauma showed high rejection rates, suggesting the influence of other factors (see above). Although statistical significance was not always reached due to small subgroup sizes, the observed trends should be considered, as the study may have been underpowered. Therefore, until these findings are confirmed in larger multicenter studies, less aggressive immunosuppressive therapy could be considered for histologically non-inflamed HSV-associated corneal scars. Conversely, consistent topical immunosuppression and close postoperative follow-up may be warranted for keratoplasty due to keratoconus- and mechanical trauma-associated scars, regardless of inflammation status.

## Data Availability

The data supporting the reported results are stored in the research database of the Freiburg Eye Center and are available on request from the principal investigator (currently D.B.).
